# Comparison of clinical and microbiological features between mucoid and non-mucoid *Pseudomonas aeruginosa* in non-cystic fibrosis bronchiectasis

**DOI:** 10.1128/spectrum.03950-25

**Published:** 2026-02-17

**Authors:** Yanghua Xiao, Tingxiu Peng, Keyi Li, Rui Zhao, Zige Wang, Guangyi Zhang

**Affiliations:** 1 Department of Respiratory and Critical Care Medicine, The First Affiliated Hospital, Jiangxi Medical College, Nanchang University 47861, Nanchang, China; 2 Jiangxi Provincial Key Laboratory of Respiratory Diseases, Jiangxi Institute of Respiratory Diseases, The First Affiliated Hospital, Jiangxi Medical College, Nanchang University 47861, Nanchang, China; 3 Department of Clinical Laboratory, Medical Center of Burn Plastic and Wound Repair, The First Affiliated Hospital, Jiangxi Medical College, Nanchang University 47861, Nanchang, China; Central Texas Veterans Health Care System, Temple, Texas, USA

**Keywords:** *Pseudomonas aeruginosa*, mucoid, resistance, tolerance, virulence

## Abstract

Mucoid *Pseudomonas aeruginosa* is frequently isolated from patients with non-cystic fibrosis bronchiectasis (NCFB), yet the clinical and microbiological consequences of this phenotype remain unclear. In this retrospective study, we analyzed 66 patients with NCFB from whom *P. aeruginosa* was isolated between 2021 and 2024 to compare mucoid and non-mucoid isolates. Among the patients, 32 harbored mucoid and 34 non-mucoid isolates. Compared with patients with non-mucoid isolates, those with mucoid isolates had poorer lung function (FEV_1_ % predicted: 52.3 ± 15.7 vs 63.8 ± 17.2; *P* = 0.006), more frequent exacerbations (2.4 ± 1.3 vs 1.5 ± 1.0 per year; *P* = 0.002), a faster annual FEV_1_ decline (−2.9% ± 2.1% vs −1.6 % ± 1.8% predicted; *P* = 0.01), more severe radiological disease, and higher treatment burden. Antimicrobial susceptibility profiles and multidrug resistance rates were similar between the groups. However, mucoid isolates showed significantly greater adhesion to airway epithelial cells but lower cytotoxicity than non-mucoid isolates. Time-kill assays revealed a higher tolerance of mucoid isolates to ceftazidime, ciprofloxacin, and tobramycin, with comparable killing observed only for colistin. RT-qPCR analysis revealed that mucoid isolates exhibited significant upregulation of alginate biosynthesis genes, alongside downregulation of motility-associated genes and T2SS/T3SS virulence genes. These findings indicate that mucoid *P. aeruginosa* infection in NCFB is linked to more severe clinical outcomes and enhanced antibiotic tolerance. Targeted therapeutic strategies addressing the unique biology of mucoid *P. aeruginosa* may be warranted in NCFB management.

**IMPORTANCE:**

Mucoid *Pseudomonas aeruginosa* is frequently isolated from patients with non-cystic fibrosis bronchiectasis (NCFB) and is associated with persistent airway infection and difficult-to-treat infections, yet it has been studied far less in NCFB than in cystic fibrosis. The study provides new insights into the biological adaptations of mucoid strains, demonstrating their capacity to persist in the airway and evade standard antimicrobial therapies. Our findings also suggest that conventional antimicrobial susceptibility testing may not fully reflect the clinical challenges posed by mucoid strains. These results highlight the need for improved diagnostic and therapeutic strategies tailored to the distinctive characteristics of mucoid *P. aeruginosa* in bronchiectasis.

## INTRODUCTION

Bronchiectasis is a chronic, progressive, and irreversible airway disease characterized by abnormal and permanent bronchial dilation, impaired mucociliary clearance, and recurrent lower respiratory tract infections (1). Non-cystic fibrosis bronchiectasis (NCFB), once considered an orphan disease, is now recognized as a significant global health burden, with an increasing prevalence in both developed and developing countries, particularly among the elderly and those with underlying respiratory conditions (2, 3). The pathogenesis of NCFB is driven by a vicious cycle of infection and inflammation that leads to progressive airway damage and impaired host defense mechanisms (4, 5).

Among the diverse pathogens implicated in NCFB, *Pseudomonas aeruginosa* is regarded as the most clinically significant because of its association with more severe disease, increased frequency of exacerbations, accelerated lung function decline, and higher risk of hospitalization and mortality (6, 7). Colonization or infection with *P. aeruginosa* is considered a turning point in bronchiectasis, often marking a transition to a more aggressive disease course (6). Once established in the airways, *P. aeruginosa* is notoriously difficult to eradicate and persists despite intensive systemic or inhaled antibiotic therapy (8, 9). This persistence is driven by the organism’s remarkable capacity for phenotypic adaptation and development of antimicrobial resistance, which collectively promotes chronic infection and therapeutic failure (10, 11). Notably, the emergence of the mucoid phenotype, which is characterized by the overproduction of the exopolysaccharide alginate, is a hallmark of chronic airway colonization and is thought to play a pivotal role in disease progression (12, 13).

Mucoid *P. aeruginosa* typically exhibits slower growth and forms smaller colonies after 24 h of incubation at 35°C, which may have contributed to these isolates being overlooked in routine culture (14). Mucoid strains are characterized by their abundant secretion of alginate, which forms a protective barrier around the *P. aeruginosa* and enhances resistance to both antibiotics and host immune defenses (15–18). This adaptation is particularly well-characterized in cystic fibrosis, where the presence of mucoid *P. aeruginosa* is strongly associated with advanced lung disease, frequent exacerbations, and poor prognosis (19, 20). However, most previous studies on bronchiectasis have focused primarily on the detection of *P. aeruginosa* as a whole, without specifically distinguishing between mucoid and non-mucoid phenotypes (6, 21). Consequently, the distinct roles of these phenotypes in disease progression and clinical management remain unclear. Moreover, the mucoid phenotype may confer additional advantages to *P. aeruginosa* beyond its well-known role in immune evasion (22). Despite the clinical significance of these features, comparative data on the clinical impact and phenotypic features of mucoid versus nonmucoid *P. aeruginosa* in NCFB are limited and inconsistent.

In this study, we aimed to comprehensively compare the clinical and microbiological characteristics of mucoid and non-mucoid *P. aeruginosa* isolates obtained from patients with NCFB. Specifically, we sought to elucidate the associations between mucoid phenotype, structural lung abnormalities, and clinical outcomes, as well as to characterize phenotypic differences that may underlie or contribute to poor prognosis.

## MATERIALS AND METHODS

### Study design and patient selection

This single-center retrospective study was conducted in the Department of Respiratory Medicine of the First Affiliated Hospital of Nanchang University between January 2021 and December 2024. We reviewed the medical records of adult patients with NCFB who attended our dedicated bronchiectasis clinic and/or were admitted to our department during the study period and had at least one positive sputum or bronchoalveolar lavage fluid culture for *P. aeruginosa*. The diagnosis of NCFB was based on recognized criteria (23) requiring compatible clinical manifestations (chronic cough, sputum production, and/or recurrent respiratory infections), together with radiological confirmation of cylindrical or cystic bronchiectasis on high-resolution computed tomography. Patients with a confirmed diagnosis of cystic fibrosis and those with incomplete clinical, radiological, lung function, or microbiological data were excluded.

### Isolate collection and identification

A total of 66 non-duplicate clinical isolates of *P. aeruginosa* were obtained from patients with NCFB (18). All isolates were collected when patients were in a stable clinical state, defined as the absence of acute exacerbation for at least 4 weeks prior to sampling. An acute exacerbation was considered present if patients reported an increased cough, sputum volume or purulence, hemoptysis, or worsening dyspnea requiring a change in treatment. Sputum and bronchoalveolar lavage fluid samples were inoculated onto blood agar plates for primary isolation and incubated aerobically at 37°C for 24–72 h. Colony identification at the species level was performed using the VITEK 2 automated identification system (bioMérieux, Marcy-l’Étoile, France) according to routine laboratory practice. Mucoid isolates were identified by smooth, viscous, glistening colonies on Luria-Bertani (LB) agar or pseudomonas isolation agar, whereas non-mucoid isolates showed dry, rough, and non-viscous colonies (24). All plates were inspected independently by two experienced microbiologists who were blinded to the clinical data. Discrepancies were resolved by consensus. The reference strain *P. aeruginosa* ATCC 27853 was used as a quality control for all the procedures.

### Antimicrobial susceptibility testing

Antimicrobial susceptibility testing was performed for all *P. aeruginosa* isolates using the broth microdilution method, in accordance with the Clinical and Laboratory Standards Institute (CLSI) guidelines (25). Briefly, bacterial suspensions were prepared in cation-adjusted Mueller-Hinton broth to a turbidity equivalent to a 0.5 McFarland standard and inoculated into 96-well microtiter plates containing serial twofold dilutions of each antibiotic. The following anti-pseudomonal agents were tested: imipenem, meropenem, ceftazidime, cefepime, piperacillin-tazobactam, ceftazidime-avibactam, cefoperazone-sulbactam, tobramycin, amikacin, ciprofloxacin, levofloxacin, aztreonam, and colistin. The plates were incubated at 37°C for 16–20 h, and the minimum inhibitory concentrations (MICs) were determined as the lowest concentration of antibiotics that completely inhibited visible growth. The results were interpreted according to CLSI breakpoints. *P. aeruginosa* ATCC 27853 was used as the quality control strain for each test batch. All susceptibility tests were performed in triplicate, and discrepant results were retested.

### Cell adhesion assays

Cell adhesion assays were performed using the human bronchial epithelial cell lines 16HBE and BEAS-2B. Both cell lines were cultured in Dulbecco’s modified Eagle’s medium (DMEM; Gibco, USA) supplemented with 10% fetal bovine serum (FBS; Gibco), 100 U/mL penicillin, and 100 μg/mL streptomycin, at 37°C in a humidified incubator with 5% CO_2_. For adhesion experiments, cells were seeded into 24-well tissue culture plates at a density of 2 × 10^5^ cells per well and allowed to reach 80–90% confluence. Overnight cultures of 10 mucoid and 10 non-mucoid *P. aeruginosa* isolates were grown in LB broth at 37°C with shaking, washed two times with phosphate-buffered saline (PBS), and resuspended in antibiotic-free DMEM. The bacterial suspensions were adjusted to a multiplicity of infection (MOI) of 10:1 (bacteria:cell). After removing the culture medium, 1 mL of the bacterial suspension was added to each well and incubated at 37°C with 5% CO_2_ for 1 h to allow bacterial adhesion. Following incubation, the wells were gently washed three times with sterile PBS to remove non-adherent bacteria. To quantify the adherent bacteria, mammalian cells and attached bacteria were lysed by adding 0.05% Triton X-100 solution (0.5 mL) for 5 min at room temperature. The lysates were serially diluted and plated on LB agar for colony enumeration. The number of adherent bacteria was calculated in colony-forming units (CFU) per well. All adhesion experiments were performed in triplicate for each strain.

### Cytotoxicity assays

Cells were seeded into 24-well plates at a density of 2 × 10^5^ cells per well and allowed to reach 80–90% confluence as previously described (26). Overnight cultures of mucoid and non-mucoid *P. aeruginosa* isolates were grown in LB broth, washed two times with PBS, and resuspended in antibiotic-free DMEM. The bacterial suspensions were adjusted to an MOI of 100:1 (bacteria:cell). After removing the culture medium, 1 mL of the bacterial suspension was added to each well and incubated for 6 h at 37°C with 5% CO_2_. After infection, cell culture supernatants were collected and centrifuged at 1,000 × *g* for 5 min to remove debris. Lactate dehydrogenase (LDH) release was measured using a commercial LDH Cytotoxicity Detection Kit (Beyotime, China), according to the manufacturer’s instructions. Absorbance was read at 490 nm using a microplate reader (BioTek, USA). Cytotoxicity was expressed as the percentage of LDH released relative to the maximum amount of LDH released (cells treated with 1% Triton X-100). All experiments were performed in triplicate.

### Time-kill assays

Time-kill assays were conducted to evaluate the bactericidal activity and antibiotic tolerance of the mucoid and non-mucoid *P. aeruginosa* isolates. Ten representative strains from each phenotype, all randomly selected and susceptible to each of the four tested antibiotics, were included in the analysis. Bacterial cultures were grown overnight in LB broth at 37°C with shaking and then diluted to approximately 1 × 10 CFU/mL in fresh cation-adjusted Mueller-Hinton broth. Clinically relevant concentrations of antibiotics were added, specifically ceftazidime (32 μg/mL), ciprofloxacin (32 μg/mL), tobramycin (32 μg/mL), and colistin (4 μg/mL). Untreated controls were included in parallel for each strain. The cultures were incubated at 37°C with shaking. At designated time points (0, 2, 4, 8, and 24 h), aliquots were removed, serially diluted in PBS, and plated onto LB agar for colony enumeration. The plates were incubated at 37°C for 18–24 h, and the number of viable bacteria (CFU/mL) was determined at each time point. All assays were performed in triplicate.

### Real-time quantitative PCR (RT-qPCR) analysis

Total RNA was extracted from planktonic cultures of mucoid and non-mucoid *P. aeruginosa* isolates in mid-logarithmic phase (OD_600_ ≈ 0.6). Bacterial cells were harvested by centrifugation at 10,000 × *g* for 5 min at 4°C and immediately resuspended in RNAprotect Bacteria Reagent (Qiagen, Germany) to stabilize RNA. Total RNA was isolated using the RNeasy Mini Kit (Qiagen, Germany) according to the manufacturer’s protocol, including on-column DNase I treatment to remove genomic DNA contamination. The RNA concentration and purity were assessed using a NanoDrop spectrophotometer (Thermo Fisher Scientific, USA). For reverse transcription, 1 μg of total RNA was converted to complementary DNA using the PrimeScript RT Reagent Kit (Takara, Japan) following the manufacturer’s instructions. RT-qPCR was performed in a 20 μL reaction volume using TB Green Premix Ex Taq II (Takara, Japan) on a QuantStudio 5 Real-Time PCR System (Applied Biosystems, USA). All reactions were performed in triplicate. Relative gene expression was calculated using the 2^−ΔΔCt^ method, with *rpoD* as the internal control. The mean fold change in gene expression was normalized to that observed in non-mucoid isolates. Primer sequences are listed in Table S1.

### Statistical analysis

All statistical analyses were performed using the SPSS software (version 26.0; IBM Corp., USA) and GraphPad Prism (version 9.0; GraphPad Software, USA). Continuous variables are expressed as mean ± standard deviation (SD) or median (interquartile range), as appropriate. Categorical variables were presented as counts and percentages. Comparisons between the two groups were performed using the Student’s *t*-test or Mann-Whitney *U*-test for continuous variables and the chi-square test or Fisher’s exact test for categorical variables. For multiple group comparisons, one-way analysis of variance (ANOVA) followed by Tukey’s post hoc test was used. Statistical significance was defined as a two-tailed *P* value of <0.05. All experiments were performed at least in triplicate, and representative data are shown. The figures were prepared using GraphPad Prism software.

## RESULTS

### Clinical characteristics of mucoid versus non-mucoid *P. aeruginosa* in NCFB

A total of 66 patients with NCFB, from whom *P. aeruginosa* was isolated, were included in this analysis, of which 32 had mucoid and 34 had non-mucoid phenotypes. The two groups were comparable in terms of age and sex distribution (Table 1), as well as the prevalence of common comorbidities such as hypertension, diabetes mellitus, ischemic heart disease, COPD, asthma, malignancy, immunosuppression, and gastroesophageal reflux disease (all *P* > 0.05). However, the mucoid group exhibited a significantly longer duration of bronchiectasis (11.2 ± 6.4 vs 7.8 ± 5.2 years, *P* = 0.008) compared to the non-mucoid group. Radiological evaluation showed that cystic bronchiectasis (43.8% vs 20.6%, *P* = 0.048) and diffuse involvement (≥3 lobes, 68.8% vs 44.1%, *P* = 0.041) were more common in the mucoid group, which also had a higher CT severity score (11.5 ± 3.2 vs 8.9 ± 3.1, *P* < 0.001), although the prevalence of predominant lower-lobe disease did not differ significantly (59.4% vs 50.0%, *P* = 0.45). In terms of lung function, patients with mucoid *P. aeruginosa* had a significantly lower FEV_1_ % predicted (52.3 ± 15.7 vs 63.8 ± 17.2, *P* = 0.006), and had lower FVC % predicted and FEV_1_/FVC ratio that did not reach statistical significance (FVC: 71.4 ± 16.9 vs 79.6 ± 17.5, *P* = 0.07; FEV_1_/FVC: 0.60 ± 0.10 vs 0.64 ± 0.09, *P* = 0.08), and were more likely to report chronic sputum production (84.4% vs 61.8%, *P* = 0.03). Regarding inflammatory markers, no significant intergroup differences were observed in the C-reactive protein level, erythrocyte sedimentation rate, blood neutrophil count, or blood eosinophil count. In contrast, sputum IL-8 levels were significantly higher in the mucoid group (*P* = 0.005), whereas differences in sputum TNF-α (*P* = 0.16) and IL-1β (*P* = 0.11) levels did not reach statistical significance.

**TABLE 1:** Comparison of clinical characteristics between mucoid and non-mucoid *P. aeruginosa* infection in patients with NCFB

Variable	Mucoid group (*n* = 32)	Non-mucoid group (*n* = 34)	*P* value
Demographics			
Age, years	63.5 ± 9.8	60.7 ± 10.3	0.21
Male sex, *n* (%)	18 (56.3)	17 (50.0)	0.62
Duration of bronchiectasis, years	11.2 ± 6.4	7.8 ± 5.2	0.008
Previous pulmonary tuberculosis, *n* (%)	5 (15.6)	4 (11.8)	0.72
Comorbidities, *n* (%)			
Hypertension	11 (34.4)	10 (29.4)	0.66
Diabetes mellitus	6 (18.8)	4 (11.8)	0.49
Ischemic heart disease	4 (12.5)	3 (8.8)	0.7
COPD	10 (31.3)	6 (17.6)	0.2
Asthma	4 (12.5)	3 (8.8)	0.7
Solid malignant tumor	3 (9.4)	2 (5.9)	0.66
Immunosuppression	5 (15.6)	2 (5.9)	0.25
Gastroesophageal reflux disease	7 (21.9)	5 (14.7)	0.41
Radiology and disease extent			
Cystic bronchiectasis, *n* (%)	14 (43.8)	7 (20.6)	0.048
Diffuse involvement (≥3 lobes), *n* (%)	22 (68.8)	15 (44.1)	0.041
Predominant lower-lobe disease, *n* (%)	19 (59.4)	17 (50.0)	0.45
CT severity score^*a*^	11.5 ± 3.2	8.9 ± 3.1	<0.001
Lung function and symptoms			
FEV_1_, % predicted	52.3 ± 15.7	63.8 ± 17.2	0.006
FVC, % predicted	71.4 ± 16.9	79.6 ± 17.5	0.07
FEV_1_/FVC ratio	0.60 ± 0.10	0.64 ± 0.09	0.08
Chronic sputum production, *n* (%)	27 (84.4)	21 (61.8)	0.03
Inflammatory markers			
C-reactive protein, mg/L	17.2 (8.9–29.8)	13.4 (6.1–21.7)	0.21
ESR, mm/h	29 (18–44)	26 (15–36)	0.37
Blood neutrophils, ×10^9^/L	5.6 ± 1.8	5.0 ± 1.7	0.19
Blood eosinophils, ×10^9^/L	0.18 ± 0.12	0.20 ± 0.13	0.55
Sputum IL-8, pg/mL	465 (320–620)	295 (180–420)	0.005
Sputum TNF-α, pg/mL	42.0 (29.0–54.2)	37.1 (24.0–48.4)	0.16
Sputum IL-1β, pg/mL	110 (75–150)	91 (61–124)	0.11

^*a*^ CT severity score calculated according to a modified Reiff score (higher scores indicate more severe disease).

### Clinical outcomes of mucoid versus non-mucoid *P. aeruginosa* in patients with NCFB

During the previous year, patients in the mucoid group had significantly worse clinical outcomes than those in the nonmucoid group (Table 2). The mean number of exacerbations was higher in the mucoid group (2.4 ± 1.3 vs 1.5 ± 1.0, *P* = 0.002), as were hospital admissions (1.2 ± 0.9 vs 0.7 ± 0.7, *P* = 0.01) and total hospital days (14.2 ± 10.5 vs 8.3 ± 9.1, *P* = 0.02). Although a greater proportion of patients with mucoid isolates were admitted to the ICU during the follow-up, the difference was not statistically significant (15.6% vs 5.9%, *P* = 0.25). The antimicrobial treatment burden was also notably greater in the mucoid group, including more frequent intravenous antibiotic courses (1.3 ± 0.9 vs 0.7 ± 0.8 per year, *P* = 0.006) and more oral antibiotic treatment days (34.8 ± 21.5 vs 21.6 ± 18.9 days/year, *P* = 0.01), as well as a higher prevalence of long-term inhaled antibiotic use (43.8% vs 20.6%, *P* = 0.04). There was no significant difference in the use of long-term macrolide therapy between groups. In terms of long-term outcomes, patients with mucoid isolates had a more rapid annual decline in FEV_1_ (−2.9% ± 2.1% vs −1.6% ± 1.8% predicted per year, *P* = 0.01). Incident long-term oxygen therapy and all-cause mortality during follow-up were more frequent in the mucoid group; however, these differences were not significant. Only one patient in the mucoid group underwent lung transplantation.

**TABLE 2:** Clinical outcomes by mucoid versus non-mucoid *P. aeruginosa* infection in patients with NCFB^*a*^

Variable	Mucoid group (*n* = 32)	Non-mucoid group (*n* = 34)	*P* value
Exacerbations and hospitalizations in the previous year			
Exacerbations in the previous year, *n*	2.4 ± 1.3	1.5 ± 1.0	0.002
Hospital admissions in the previous year, *n*	1.2 ± 0.9	0.7 ± 0.7	0.01
Hospital days in the previous year, days	14.2 ± 10.5	8.3 ± 9.1	0.02
Ever admitted to ICU during follow-up, *n* (%)	5 (15.6)	2 (5.9)	0.25
Antimicrobial treatment burden			
Intravenous antibiotic courses in the previous year, courses/year	1.3 ± 0.9	0.7 ± 0.8	0.006
Oral antibiotic days in the previous year, days/year	34.8 ± 21.5	21.6 ± 18.9	0.01
Long-term inhaled antibiotics, *n* (%)	14 (43.8)	7 (20.6)	0.04
Long-term macrolide therapy, *n* (%)	11 (34.4)	7 (20.6)	0.21
Long-term outcomes			
FEV_1_ decline, % predicted per year	−2.9 ± 2.1	−1.6 ± 1.8	0.01
Incident long-term oxygen therapy, *n* (%)	6 (18.8)	3 (8.8)	0.29
All-cause mortality during follow-up, *n* (%)	4 (12.5)	2 (5.9)	0.41
Lung transplantation during follow-up, *n* (%)	1 (3.1)	0 (0)	0.47

^*a*^ Exacerbation, hospitalization, and antibiotic use refer to events recorded during the 12 months preceding the index sputum or BALF cultures.

### Associations of mucoid *P. aeruginosa* phenotype with clinical characteristics and outcomes

In patients with NCFB, longer disease duration, cystic changes, and worse lung function were independently associated with the presence of a mucoid *P. aeruginosa* phenotype (Table 3). In multivariable logistic regression, duration of bronchiectasis remained significantly associated with mucoid phenotype (adjusted odds ratio [aOR]: 1.48 per 5-year increase, 95% confidence interval [CI]: 1.07–2.05; *P* = 0.018), as did cystic bronchiectasis (aOR: 3.05, 95% CI: 1.03–9.07; *P* = 0.044) and lower baseline FEV_1_ % predicted (aOR: 1.39 per 10% decrease, 95% CI: 1.01–1.92; *P* = 0.042). Age, sex, diffuse involvement, and CT severity score were not independently associated with the mucoid phenotype. Mucoid *P. aeruginosa* infection/colonization was independently associated with poor clinical outcome (Table 4). After adjustment for age, duration of bronchiectasis, baseline FEV_1_ and CT severity score, patients with mucoid *P. aeruginosa* had higher odds of frequent exacerbations (≥2/year) than those with non-mucoid *P. aeruginosa* (aOR: 2.45, 95% CI: 1.12–5.39; *P* = 0.024). In linear regression, mucoid phenotype was related to a higher number of hospital admissions in the previous year (β: 0.37 admissions/year, 95% CI: 0.12–0.62; *P* = 0.005) and a faster decline in FEV₁ (β: −1.03% predicted per year, 95% CI: −1.70 to −0.36; *P* = 0.003), independent of other covariates. Among the covariates, a higher CT severity score was also associated with greater odds of frequent exacerbations (aOR: 1.12 per 1-point increase, 95% CI: 1.01–1.25; *P* = 0.037) and more annual hospital admissions (β: 0.09, 95% CI: 0.01–0.17; *P* = 0.029).

**TABLE 3:** Multivariable analysis of factors associated with mucoid *P. aeruginosa* phenotype

Variable	Adjusted OR (95% CI)	*P* value
Age, per 10-year increase	1.10 (0.81–1.49)	0.54
Male sex	1.25 (0.47–3.33)	0.66
Duration of bronchiectasis, per 5-year increase	1.48 (1.07–2.05)	0.018
Cystic bronchiectasis	3.05 (1.03–9.07)	0.044
Diffuse involvement (≥3 lobes)	1.63 (0.57–4.70)	0.36
Baseline FEV_1_ % predicted, per 10% decrease	1.39 (1.01–1.92)	0.042
CT severity score, per 1-point increase	1.09 (0.96–1.23)	0.19

**TABLE 4:** Multivariable associations between mucoid phenotype and major clinical outcomes^*a*^

Outcome	Variable	Adjusted OR/*β* (95% CI)	*P* value
Frequent exacerbations (≥2/year)	Mucoid phenotype	2.45 (1.12 to 5.39)	0.024
	Age, per year	1.03 (0.98 to 1.07)	0.19
	Duration of bronchiectasis, per year	1.07 (0.99 to 1.15)	0.07
	Baseline FEV_1_ % predicted	0.98 (0.96 to 1.00)	0.09
	CT severity score	1.12 (1.01 to 1.25)	0.037
Annual hospital admissions	Mucoid phenotype	0.37 (0.12 to 0.62)	0.005
	Age, per year	0.02 (–0.01 to 0.05)	0.23
	Baseline FEV_1_ % predicted	–0.01 (–0.03 to 0.01)	0.14
	CT severity score	0.09 (0.01 to 0.17)	0.029
FEV_1_ decline, % predicted per year	Mucoid phenotype	–1.03 (–1.70 to –0.36)	0.003
	Age, per year	–0.02 (–0.08 to 0.04)	0.51
	Duration of bronchiectasis, per year	–0.03 (–0.12 to 0.06)	0.53
	Baseline FEV_1_ % predicted	–0.02 (–0.05 to 0.01)	0.23

^*a*^ β, regression coefficient; CI, confidence interval; CT, computed tomography; FEV, forced expiratory volume in 1 s; OR, odds ratio. Logistic regression was used to model frequent exacerbations (≥2/year), and results are presented as adjusted odds ratios (aORs) with 95% CIs. Linear regression models were used for annual hospital admissions and FEV decline, and the results were expressed as β coefficients with 95% CIs.

### Antimicrobial susceptibility and multidrug resistance profiles

To determine whether the worse clinical outcomes in patients with mucoid *P. aeruginosa* were attributable to differences in antimicrobial susceptibility, we compared the susceptibility profiles of mucoid and non-mucoid isolates. MIC data for all isolates are provided in Table S2. There were no significant differences in antimicrobial susceptibility between the two groups (Table 5). The proportions of carbapenem resistance were similar in the two groups, with 40.6% mucoid and 44.1% non-mucoid isolates resistant to imipenem (*P* = 0.77) and 43.8% versus 41.2% resistant to meropenem (*P* = 0.83). Resistance to cephalosporins, including ceftazidime (43.8% vs 26.5%, *P* = 0.14) and cefepime (18.8% vs 11.8%, *P* = 0.51), did not differ significantly between the groups. The prevalence of resistance to aminoglycosides, β-lactam/β-lactamase inhibitor combinations, fluoroquinolones, monobactam, and colistin was also low and comparable between mucoid and non-mucoid isolates. Furthermore, the rates of multidrug-resistant (MDR) and extensively drug-resistant (XDR) phenotypes were similar in both groups (MDR: 18.8% vs 20.6%, *P* = 0.86; XDR: 6.3% vs 5.9%, *P* = 1.00). These findings indicate that the mucoid phenotype is not associated with increased antimicrobial resistance or a higher prevalence of MDR/XDR *P. aeruginosa*.

**TABLE 5:** Antimicrobial susceptibility profiles and multidrug resistance among mucoid and non-mucoid *P. aeruginosa* isolates

Antimicrobial agent/phenotype	Mucoid group (*n* = 32), *n* (%)	Non-mucoid group (*n* = 34), *n* (%)	*P* value
Carbapenems			
Imipenem resistant	13 (40.6)	15 (44.1)	0.77
Meropenem resistant	14 (43.8)	14 (41.2)	0.83
Cephalosporins			
Ceftazidime resistant	14 (43.8)	9 (26.5)	0.14
Cefepime resistant	6 (18.8)	4 (11.8)	0.51
Aminoglycosides			
Amikacin resistant	1 (3.1)	2 (5.9)	1
Tobramycin resistant	0 (0)	1 (2.9)	1
β-Lactam/β-lactamase inhibitors			
Piperacillin-tazobactam resistant	3 (9.4)	3 (8.8)	1
Ceftazidime-avibactam resistant	3 (9.4)	4 (11.8)	1
Cefoperazone-sulbactam resistant	1 (3.1)	3 (8.8)	0.62
Fluoroquinolone			
Ciprofloxacin resistant	12 (37.5)	13 (38.2)	0.95
Monobactam			
Aztreonam resistant	4 (12.5)	3 (8.8)	0.7
Polymyxin			
Colistin resistant	0 (0)	1 (2.9)	1
Composite resistance phenotypes			
MDR (multidrug-resistant)	6 (18.8)	7 (20.6)	0.86
XDR (extensively drug-resistant)	2 (6.3)	2 (5.9)	1

### Mucoid isolates exhibit higher antibiotic tolerance compared to non-mucoid isolates

**Fig 1:**
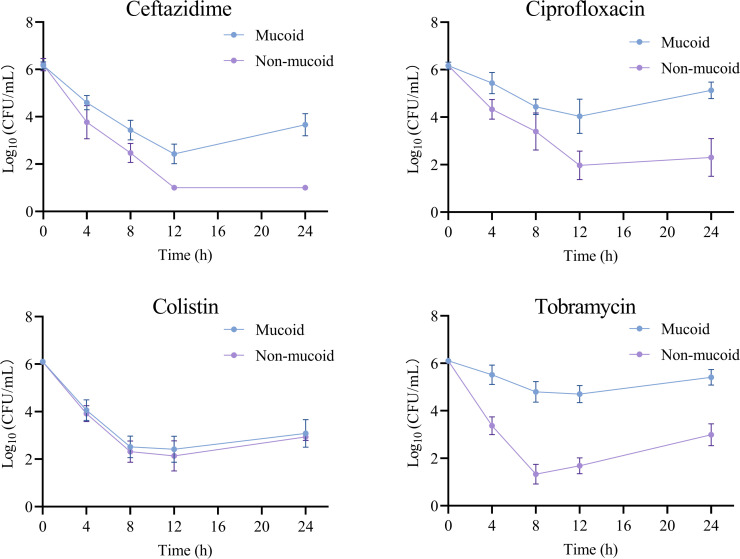
Time-kill kinetics of mucoid and non-mucoid *P. aeruginosa* isolates exposed to antipseudomonal antibiotics. Bacterial survival was measured as log_10_ CFU/mL at 0, 4, 8, 12, and 24 h post-exposure. Data are shown as mean ± SD from 10 independent mucoid and 10 independent non-mucoid isolates.

To assess antibiotic tolerance, time-kill assays were performed for mucoid and non-mucoid *P. aeruginosa* isolates, using four clinically relevant antipseudomonal agents. As shown in Fig. 1, the mucoid isolates consistently showed attenuated bactericidal responses to most of the antibiotics tested. For ceftazidime, non-mucoid isolates exhibited rapid and sustained killing, with a >4-log_10_ reduction at 12 h and near-complete eradication by 24 h, whereas mucoid isolates showed only a 2–3 log_10_ reduction at 12 h followed by regrowth at 24 h. A similar pattern was observed with ciprofloxacin. Non-mucoid isolates declined to approximately 2 log_10_ CFU/mL at 12 h and remained low at 24 h, while mucoid isolates showed a slower, less pronounced decrease, stabilizing above 4 log_10_ CFU/mL at 12 h with subsequent regrowth by 24 h. In the presence of tobramycin, non-mucoid isolates demonstrated a >4-log_10_ reduction within 12 h, which was maintained for 24 h, whereas mucoid isolates showed only a 2–3 log_10_ decline and substantial regrowth at 24 h. By contrast, colistin produced similar killing kinetics in both groups, with an initial 3–4 log_10_ reduction by 8 h followed by partial regrowth at 24 h and no significant differences between mucoid and non-mucoid isolates.

### Mucoid isolates exhibit enhanced adhesion but reduced cytotoxicity compared to non-mucoid isolates

**Fig 2:**
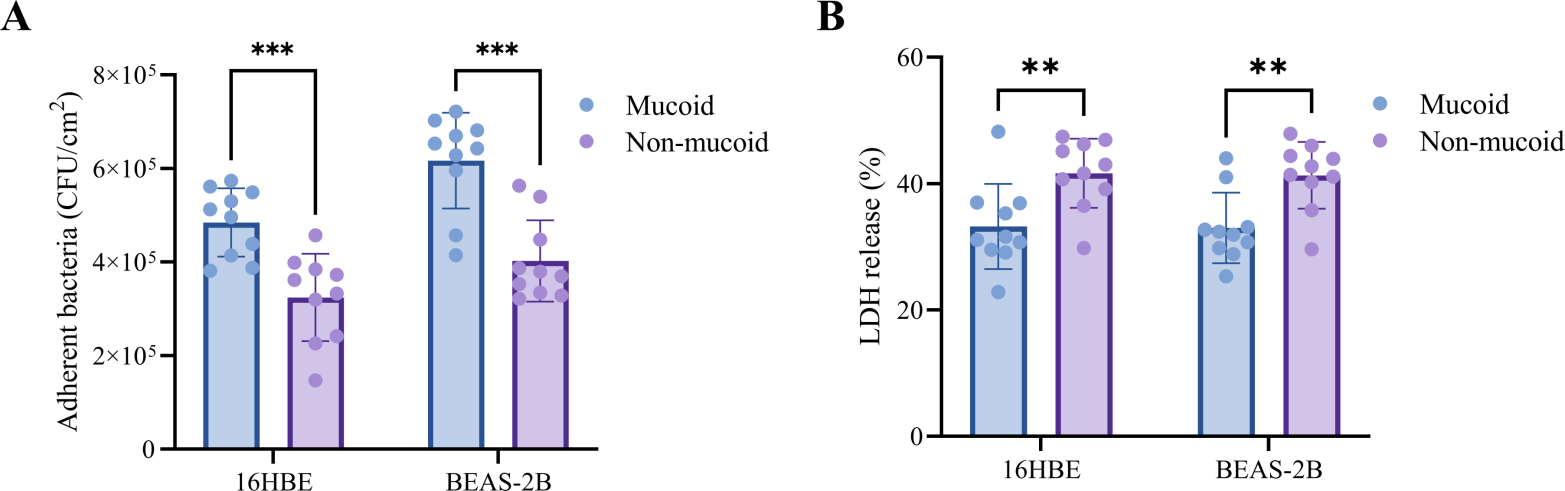
Comparison of adhesion and cytotoxicity between mucoid and non-mucoid *P. aeruginosa* isolates. (**A**) Quantification of adherent bacteria to 16HBE and BEAS-2B cells after 1 h of incubation. Adherent bacteria were enumerated as colony-forming units per cm^2^ (CFU/cm^2^). (**B**) Cytotoxicity of mucoid and non-mucoid isolates toward 16HBE and BEAS-2B cells was assessed by measuring lactate dehydrogenase (LDH) release at 6 h post-infection. Each data point represents an individual isolate; bars indicate mean ± standard deviation. Statistical significance was determined by an unpaired *t*-test (**P < 0.01, ***P < 0.001).

To compare the adhesive and cytotoxic properties of mucoid and non-mucoid *P. aeruginosa* isolates, we performed *in vitro* assays using the 16HBE and BEAS-2B cell lines. In adhesion assays, mucoid isolates showed significantly higher attachment to both cell types than non-mucoid isolates, although the magnitude of the difference was modest (Fig. 2A). The mean number of adherent bacteria on 16HBE cells was 474,100 ± 75,900 CFU/cm^2^ for mucoid isolates and 321,800 ± 70,600 CFU/cm^2^ for non-mucoid isolates (1.47-fold increase, *P* < 0.001). Similarly, in BEAS-2B cells, mucoid isolates displayed an average adhesion of 636,900 ± 94,800 CFU/cm^2^, which was significantly greater than the 400,700 ± 81,600 CFU/cm^2^ observed in non-mucoid isolates (1.59-fold increase, *P* < 0.001). In contrast, assessment of cytotoxicity by LDH release at 6 h post-infection revealed that mucoid isolates induced significantly less damage in both cell lines (Fig. 2B). The percentage of LDH release from 16HBE cells was 34.2% ± 7.0% for mucoid isolates compared to 41.6% ± 6.1% for non-mucoid isolates (*P* < 0.01), and for BEAS-2B cells, the LDH release was 33.0% ± 5.1% for mucoid isolates versus 40.7% ± 6.4% for non-mucoid isolates (*P* < 0.01).

### Differential expression of alginate, motility, virulence, and resistance genes in mucoid and non-mucoid *P. aeruginosa*

**Fig 3:**
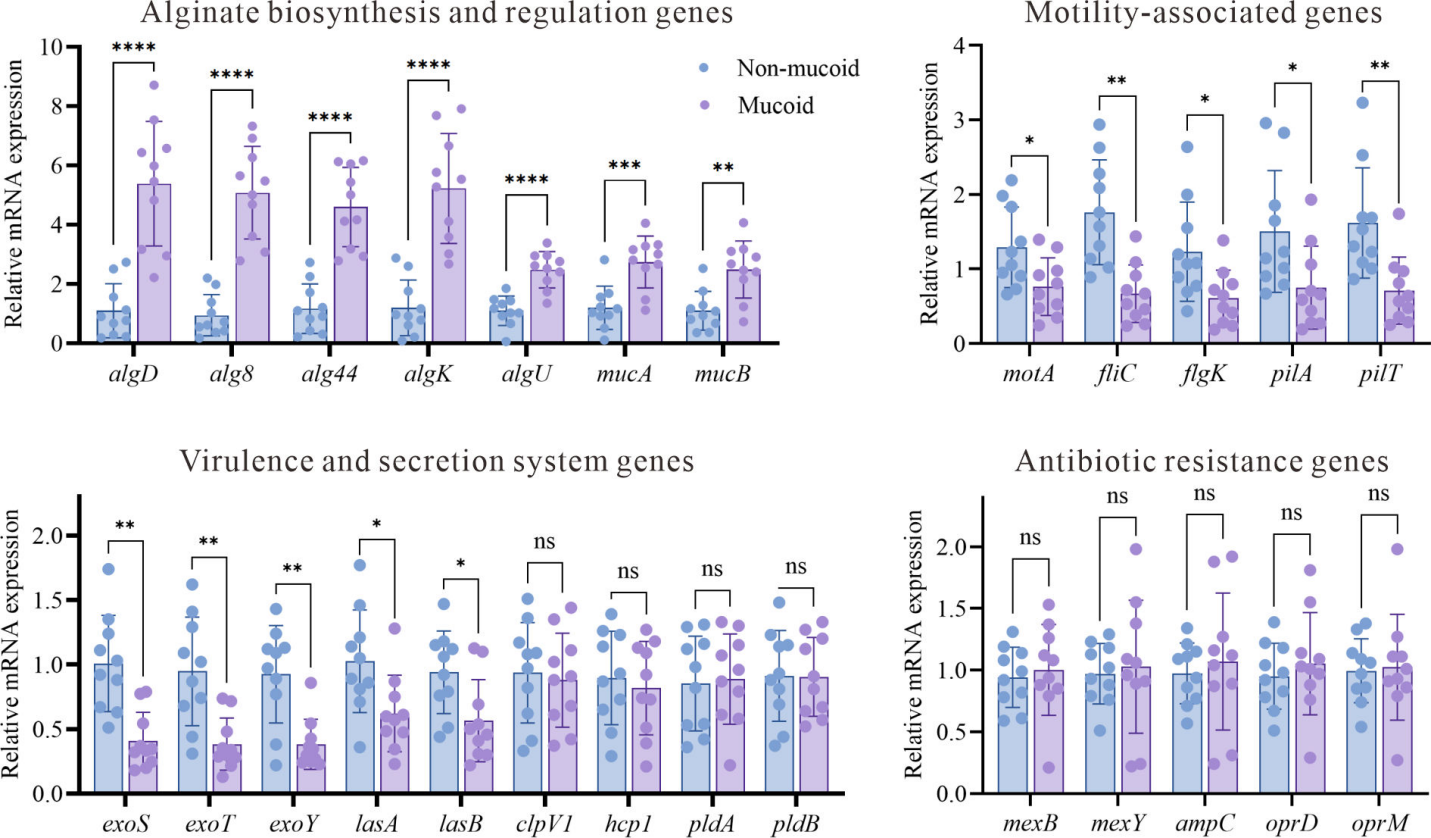
Differential expression of genes associated with alginate biosynthesis, motility, virulence, and antibiotic resistance in mucoid and non-mucoid *P. aeruginosa* isolates. Expression values were normalized to housekeeping gene *rpoD* and are shown relative to the mean of the non-mucoid group. Each data point represents an individual isolate; bars indicate mean ± SD. Statistical significance was determined by an unpaired *t*-test: ns, not significant; *P < 0.05; **P < 0.01; and ****P < 0.0001.

To investigate the molecular basis underlying the distinct phenotypes of mucoid and non-mucoid *P. aeruginosa* isolates, we compared the expression levels of key genes related to alginate biosynthesis and regulation, motility, virulence, and antibiotic resistance between the two groups using RT-qPCR. As shown in Fig. 3, mucoid isolates exhibited markedly increased expression of alginate biosynthesis and regulatory genes, including *algD* (5.4-fold), *alg8* (5.1-fold), *alg44* (4.6-fold), *algK* (5.2-fold), *algU* (2.5-fold), *mucA* (2.7-fold), and *mucB* (2.5-fold), compared to non-mucoid isolates (all *P* < 0.01). In contrast, the expression of motility-associated genes (*motA*, *fliC*, *flgK*, *pilA*, and *pilT*) was significantly downregulated in mucoid isolates, indicating reduced transcription of flagellar and type IV pili genes. Similarly, several major virulence and secretion system genes were significantly downregulated in mucoid isolates compared to non-mucoid isolates. Notably, the expression of genes encoding the type III secretion system (T3SS) effectors *exoS* (0.41-fold), *exoT* (0.38-fold), and *exoY* (0.38-fold) was markedly reduced (all *P* < 0.01). In addition, the expression levels of the type II secretion system (T2SS) genes *lasA* (0.62-fold) and *lasB* (0.56-fold) were significantly decreased (*P* < 0.05). In contrast, the type VI secretion system genes (*clpV1* and *hcp1*) and phospholipase genes (*pldA* and *pldB*) were not significantly different (*P* > 0.05). However, the expression of key antibiotic resistance and efflux pump genes, including *mexB*, *mexY*, *ampC*, *oprD*, and *oprM*, remained largely unchanged between mucoid and non-mucoid isolates (*P* > 0.05).

## DISCUSSION

Consistent with prior observations in cystic fibrosis populations, the emergence of mucoid *P. aeruginosa* is a well-established marker of disease progression and poor outcome (27, 28). Similarly, our study demonstrated that, in patients with NCFB infected with *P. aeruginosa*, a mucoid phenotype was associated with more severe structural lung disease, worse lung function, and poorer clinical outcomes. Notably, these associations were observed despite broadly comparable antimicrobial susceptibility profiles between mucoid and non-mucoid isolates, suggesting that factors beyond conventional resistance mechanisms may contribute to the poor clinical course.

Importantly, the presence of mucoid *P. aeruginosa* is independently associated with adverse short-term and long-term outcomes. Patients with mucoid isolates experienced more frequent exacerbations, more hospital admissions and days in hospital, greater exposure to intravenous and oral antibiotics, and a faster decline in FEV_1_. These associations persisted after adjustment for age, baseline FEV_1_, duration of bronchiectasis, and CT severity score. The association between mucoid *P. aeruginosa* and higher odds of frequent exacerbations mirrors findings from prior studies linking *P. aeruginosa* infection in general to poor outcomes in bronchiectasis (21, 29), suggesting that not all *P. aeruginosa* infections carry equal prognostic weights. In this context, mucoidy may help refine risk stratification among *P. aeruginosa*-infected NCFB patients, which is increasingly being recognized as a key objective in bronchiectasis management (20). Although trends were observed toward higher rates of ICU admission, incident long-term oxygen therapy, and mortality in the mucoid group, they did not reach statistical significance, most likely reflecting the limited statistical power given the sample size and follow-up duration. Nonetheless, the clearly worse exacerbation profile, hospitalization burden, and accelerated decline in lung function emphasize the clinical relevance of distinguishing mucoid from nonmucoid *P. aeruginosa* in routine practice. Functionally, the presence of mucoid *P. aeruginosa* correlated with lower FEV_1_ % predicted and a faster annual decline in FEV_1_. The association between mucoid phenotype and accelerated FEV_1_ decline, independent of baseline FEV_1_ and CT severity, suggests that mucoid *P. aeruginosa* may contribute directly to functional deterioration, which is consistent with data linking chronic *P. aeruginosa* infection to lung function decline in bronchiectasis (21, 30). Interestingly, the levels of inflammatory markers (CRP, ESR, blood neutrophils, and eosinophils) did not differ between the mucoid and non-mucoid groups, whereas the sputum IL-8 levels were significantly higher in the mucoid group. IL-8 is a key chemokine that mediates neutrophil recruitment to the airways and has been linked to bacterial load and clinical severity in bronchiectasis (31, 32). The selective elevation of sputum IL-8 suggests localized, chronic neutrophilic airway inflammation in patients with mucoid *P. aeruginosa* and may contribute to the gradual yet progressive deterioration observed in these patients.

Despite markedly worse clinical outcomes and greater antibiotic exposure, mucoid and non-mucoid isolates in our cohort exhibited broadly comparable *in vitro* antimicrobial susceptibility profiles for multiple classes of antibiotics. This is in contrast with the findings of Wu et al., who recently reported that clinically isolated mucoid *P. aeruginosa* strains showed substantially lower resistance rates than non-mucoid strains (33). A key reason for this discrepancy is likely the difference between the study populations. Our analysis was restricted to *P. aeruginosa* isolates obtained from the respiratory tract of patients with stable NCFB, whereas Wu et al. included isolates from a wide range of clinical sources, including urine, drainage fluid, and skin swabs, and did not limit their inclusion by underlying disease type. Efflux pumps are one of the major mechanisms that contribute to antibiotic resistance in *P. aeruginosa* (34). However, the prevalence of MDR and XDR phenotypes in our study was low and did not differ between the groups. These data suggest that classical resistance, conventionally defined by MIC-based susceptibility testing, is unlikely to be the main driver of poor outcomes in patients with mucoid *P. aeruginosa*.

In contrast, our time-kill assays clearly demonstrated that mucoid isolates exhibited increased antibiotic tolerance. For ceftazidime, ciprofloxacin, and tobramycin, mucoid isolates showed attenuated bactericidal responses, with smaller log reductions at early time points and substantial regrowth by 24 h, whereas non-mucoid isolates underwent rapid and sustained killing. The killing kinetics of colistin were comparable between the two groups. These findings suggest that mucoid bacterial populations can endure prolonged antibiotic exposure without necessarily acquiring classical resistance mutations, reflecting tolerance and persistence rather than elevated resistance. This observation is highly relevant to clinical practice, as antibiotic tolerance is increasingly recognized as a contributor to treatment failure, recurrent infection, and chronic airway colonization (35, 36). The mucoid phenotype may confer a survival advantage during antibiotic therapy, which likely contributes to persistent infection and may account for the paradoxically poor clinical outcomes observed, even in the absence of obvious antimicrobial resistance. Notably, this increased tolerance was not accompanied by the upregulation of classic resistance or efflux pump genes (*mexB*, *mexY*, *ampC*, *oprD*, and *oprM*), implying that the increased antibiotic tolerance observed in mucoid *P. aeruginosa* is primarily attributable to the upregulation of alginate-related genes rather than alterations in the expression of major efflux pump systems. This pattern of gene expression likely contributes to the persistent and recalcitrant nature of mucoid *P. aeruginosa* infections in patients with NCFB. In the setting of mucoid *P. aeruginosa* infections, treatment strategies that prioritize the optimization of drug delivery, including inhaled formulations and higher local antibiotic concentrations, together with prolonged or combination regimens and active disruption of biofilm structure, may be more important than simply escalating to broader-spectrum systemic agents (37).

*In vitro* adhesion and cytotoxicity assays further elucidated the pathobiology of the mucoid *P. aeruginosa*. Mucoid isolates exhibited significantly higher adhesion to both 16HBE and BEAS-2B epithelial cells while inducing less LDH release than non-mucoid isolates. This phenotype, characterized by enhanced adhesion but reduced acute cytotoxicity, suggests that mucoid *P. aeruginosa* is adapted to stable, long-term colonization of the airway epithelium, rather than acute epithelial destruction. Alginate and other exopolysaccharides can promote bacterial attachment and persistence while shielding organisms from host defenses and antimicrobial agents (38, 39). Gene expression profiling provided a mechanistic explanation for these findings. Mucoid isolates showed a marked upregulation of alginate biosynthesis genes. However, the expression of major virulence determinants, particularly type III secretion system effectors (*exoS*, *exoT*, and *exoY*) and type II secretion system proteases (*lasA* and *lasB*), was significantly reduced in the mucoid isolates. One possible mechanism is that loss-of-function mutations in *mucA*, commonly reported in mucoid clinical isolates, constitutively activate AlgU, thereby promoting alginate biosynthesis while repressing acute-phase virulence programs, including the T3SS (40). In addition, T3SS gene expression is sensitive to strain background and environmental cues (41), which may contribute to the observed inter-isolate variability. T3SS effectors and elastolytic proteases are central mediators of acute cytotoxicity, epithelial injury, and immune evasion in acute *P. aeruginosa* infections (42, 43). This molecular adaptation may allow mucoid *P. aeruginosa* to evade immune detection and establish long-term colonization, thereby perpetuating airway inflammation and structural damage (44). Notably, type VI secretion system genes (*clpV1* and *hcp1*) and phospholipase genes (*pldA* and *pldB*) did not differ significantly between groups, suggesting that not all virulence systems were uniformly downregulated and that mucoid *P. aeruginosa* may retain capabilities relevant to interbacterial competition and niche maintenance within the polymicrobial airway environment. Overall, these data suggest that mucoid *P. aeruginosa* is more consistent with an adhesive and relatively noncytotoxic phenotype than with a planktonic, acutely virulent state. This phenotype is characterized by enhanced production of alginate and downregulation of major secreted virulence determinants, which together may favor chronic infection, sustained airway inflammation, and progressive lung damage.

However, this study has several limitations. First, this was a retrospective single-center study, and the cohort was predominantly composed of older adults, which may limit generalizability to younger patients and other geographic settings. Although we included NCFB patients over nearly 4 years, the sample size was relatively modest and may have limited our ability to detect smaller effect sizes, particularly for infrequent outcomes such as ICU admissions, long-term oxygen therapy, transplantation, and mortality. Second, only one isolate per patient was analyzed, which precludes assessment of within-patient temporal evolution of mucoid conversion. Third, although the association between alginate overproduction and biofilm development is well established, we did not directly assess biofilm architecture in this isolate set. Future studies using standardized, airway-relevant biofilm models would help to further contextualize our findings.

### Conclusion

This study demonstrated that mucoid *P. aeruginosa* in NCFB was associated with more severe clinical manifestations, enhanced antibiotic tolerance, and a distinct molecular signature characterized by increased adhesion and reduced acute virulence. These findings highlight the inadequacy of relying solely on conventional resistance testing in the management of chronic *P. aeruginosa* infections and emphasize the need for novel strategies targeting the unique biology of the mucoid phenotype. Integrating phenotypic and molecular profiling into clinical algorithms may improve the risk stratification and guide the development of targeted therapeutics.

## Data Availability

Data will be made available on request.

## SUPPLEMENTAL MATERIAL

Table S1 spectrum.03950-25-s0001.xlsx Primers used in this study.

Table S2 spectrum.03950-25-s0002.xlsx MICs of tested antibiotics for *Pseudomonas aeruginosa* isolates included in this study (mucoid and non-mucoid).

ASM does not own the copyrights to Supplemental Material that may be linked to, or accessed through, an article. The authors have granted ASM a non-exclusive, world-wide license to publish the Supplemental Material files. Please contact the corresponding author directly for reuse.
